# High-Dose Hook Effect in 17-Hydroxyprogesterone Assay in a Patient with 21-Hydroxylase Deficiency

**DOI:** 10.4274/jcrpe.2180

**Published:** 2015-12-03

**Authors:** Mesut Parlak, Hamit Yaşar Ellidağ, Doğa Türkkahraman

**Affiliations:** 1 Antalya Education and Research Hospital, Clinic of Pediatric Endocrinology, Antalya, Turkey; 2 Antalya Education and Research Hospital, Clinic of Biochemistry, Antalya, Turkey

**Keywords:** High-dose hook effect, 17-hydroxyprogesterone, immunoassay, congenital adrenal hyperplasia

## Abstract

Congenital adrenal hyperplasia (CAH) describes a group of disorders characterized by enzyme defects in adrenal steroidogenesis. 21-hydroxylase deficiency (21-OHD) is the most commonly encountered form. The analysis of steroids in pediatric cases requires high-sensitivity assays. A 14-year-old Syrian girl was referred for evaluation of short stature, amenorrhea, and hirsutism. On physical examination, breast development was Tanner stage 1. She had a phallic clitoris with a single urogenital orifice. Laboratory findings revealed primary adrenal deficiency with high androgen levels and low levels of 17-hydroxyprogesterone (17-OHP), (<0.05 ng/mL) and estrogen. This unexpected result led to suspicion of a high-dose hook effect. The measurement was repeated after 1/10 dilution of serum, and a high level of 17-OHP (115.4 ng/mL) was detected with the same test-enzyme-linked immunosorbent assay (ELISA). Simple virilizing form of CAH (21-OHD) was suspected and confirmed with genetic analysis. After initiation of glucocorticoid therapy, breast development was noted along with a decrease in testosterone level and an increase in estrogen level. To our knowledge, this is the first case report of hook effect for 17-OHP immunoassay in a patient with 21-OHD. High-dose hook effect should be suspected in patients with CAH when the test results are incompatible with one another. Additionally, this case demonstrates that a high testosterone level can block aromatase activity and consequently also estrogen production and breast development.

**WHAT IS ALREADY KNOWN ON THIS TOPIC?**

Congenital adrenal hyperplasia (CAH) describes a group of disorders characterized by enzyme defects in adrenal steroidogenesis. In laboratory management, false-negative results can occur at extremely high level of substrate in the assay systems.

**WHAT THIS STUDY ADDS?**

This is the first case report of hook effect for 17-hydroxyprogesterone immunoassay in a patient with 21-hydroxylase deficiency. High-dose hook effect should be suspected in patients with CAH when the test results are incompatible with each other.

## INTRODUCTION

Congenital adrenal hyperplasia (CAH) describes a group of autosomal recessive disorders characterized by enzyme defects in adrenal steroidogenesis. 21-hydroxylase deficiency (21-OHD) is the most frequently encountered form and constitutes 90-95% of the cases. CAH is clinically classified into classical and non-classical forms. Classical CAH includes the salt-wasting (SW) form with complete lack of enzyme activity and the simple virilizing (SV) form with partial lack of enzyme activity. Non-classical (late-onset) form is caused by a mild deficiency of 21-OH enzyme activity that presents later in life (1). To date, more than 100 mutations have been reported in the human CYP21A2 gene. Approximately 95% of all disease-causing mutations in CYP21A2 gene are large deletions, large conversions, or point mutations (2).

The analysis of steroids in pediatric cases requires high-sensitivity assays. False-negative results can occur at extremely high level of substrate in the assay systems. This is called high- dose hook effect (3). It is well-known that the high-dose hook effect may give falsely low results for several hormones such as prolactin (4), growth hormone (5), thyroid stimulating hormone (TSH) (6), thyroglobulin (7), gonadotropins [luteinizing hormone (LH) and follicle-stimulating hormone (FSH)] (8), testosterone (9), human chorionic gonadotropin (hCG) (10), calcitonin (11), and aldosterone (12), however, it has never been described for 17-hydroxyprogesterone (17-OHP).

Here, we report that high-dose hook effect led to undetectable level of 17-OHP in a patient with SV form of CAH caused by a previously known p.I173N mutation.

## CASE REPORT

A 14-year-old Syrian girl was referred to our pediatric endocrinology clinic for evaluation of short stature, amenorrhea, and hirsutism. In her medical history, there was no known consanguinity between the parents. She had three healthy sisters without hirsutism and/or clitoromegaly. On physical examination, height was 140.5 cm [Standard deviation score (SDS): -3.5] and weight was 43.4 kg (Body mass index (BMI): 22.1, +0.8 SDS). Blood pressure was normal. She appeared to be of normal intelligence. Target height was 153.5 cm (SDS: -1.0). The patient’s breast development conformed to Tanner stage 1 and her pubic hair to Tanner stage 5. Facial acne, severe hirsutism with systemic skin hyperpigmentation, and clitoromegaly (4x1.5 cm) with a single urogenital orifice (Prader stage 4) were detected. There was no palpable gonad in the inguinal region.

Laboratory findings revealed a LH level of 3.1 mIU/mL (N: 2.1-10.8) and a FSH level of 6.3 mIU/mL (N: 4.5-22). Estradiol level was <20 pg/mL, progesterone 31.9 ng/mL (N: 0.1-1.5), prolactin 12.6 ng/mL (N: 2.7-19.6), free thyroxine (fT4) 0.8 ng/mL (N: 0.6-1.4), and TSH 1.7 uIU/mL (N: 0.3-4.6). Serum sodium level was 138 mmol/L (N: 136-146) and potassium was 4.3 mmol/L (N: 3.5-5.1). Adrenocorticotropic hormone (ACTH) level was 319 pg/mL (N: 4.7-48.8), cortisol 7.8 ug/dL (N: 6.7-22.6), dehydroepiandrosterone-sulfate (DHEA-S) 720.6 µg/dL (N: 35-430), total testosterone 4.4 ng/mL (N: 0.1-0.7), androstenedione 13 ng/mL (N: 0.3-3.3), and 17-OHP level was below the detection limit (<0.05 ng/mL), (N: 0.2-1.3) (Table 1).

ACTH1-24 stimulated levels for 17-OHP, cortisol, and DHEA-S were <0.05 ng/mL, 8.03 ug/dL, and 750 ug/dL, respectively. The epiphyses were closed and the bone age was adult level. Pelvic ultrasonography confirmed the presence of a uterus (53x13x22 mm) with bilateral ovaries (2 mL/2.3 mL). Adrenal ultrasonography revealed adrenal hyperplasia. Karyotype was 46,XX.

Surprisingly, initial and stimulated serum 17-OHP levels were very low, while the androgen levels were high. This unexpected result led to suspicion of a high-dose hook effect. The measurement was repeated after 1/10 dilution of serum, and a very high level of basal 17-OHP (115.4 ng/mL) was detected with the same test-enzyme-linked immunosorbent assay (ELISA) (DiaMetra, Segrate, Italy). The assay employs the quantitative sandwich enzyme immunoassay technique.

These findings were in line with a diagnosis of the SV form of 21-OHD, and oral hydrocortisone (15 mg/m2/day) therapy was initiated. Two months later, the serum levels of androgens were still above normal limits (Table 1) and, therefore, oral hydrocortisone was switched to oral dexamethasone (0.5 mg/day). At the clinical follow-up on this therapy, breast tissue progressed to Tanner stage 4 in three months. Clinical and laboratory findings at follow-up are shown in Table 1. After psychological evaluation, vaginoplasty and cliteroplasty were scheduled.

To confirm the diagnosis of 21-OHD, genetic analysis was performed from the DNA of peripheral blood leucocytes of the patient, and a previously known (13) homozygous missense p.I173N (c.518T>A) mutation in exon 4 of CYP21A2 gene was detected. Genetic analysis could not be performed in the parents due to economic reasons.

## DISCUSSION

CAH has a wide spectrum of clinical severity depending upon the residual enzyme activity. Due to enzyme deficiency, steroid precursors accumulate in the adrenal cortex and shunt through the adrenal androgen biosynthetic pathway. Increased synthesis of adrenal androgens causes virilization (1,2). Girls with the SV form may show ambiguous external genitalia at birth and therefore are diagnosed earlier compared to boys. In later years, excessive synthesis of adrenal androgens causes hirsutism, advanced bone age, and finally short stature. In this report, we present a late-diagnosed girl with SV form of CAH (21-OHD) caused by a homozygous mutation. Homozygous p.I173N (c.518T>A) mutation has been previously reported in patients with SV and SW forms of 21-OHD (13).

The analysis of steroids in pediatric cases requires methods of high sensitivity. Different biochemical techniques such as gas chromatography, liquid chromatography, and immunoassays including ELISA and immunoradiometric assay (IRMA) have been used to determine serum steroid levels. Immunoassays have evolved because of their adequate specificity and accuracy. However, in the diagnosis of CAH in the neonatal period, immunoassays might stumble because of the high levels of steroid precursors which might cause cross-reactivity. The best method in that situation is liquid chromatography/mass spectrometry which gives more precise and accurate results.

In sandwich immunoassays, as the concentration of analyte increases above a certain point, the system gets saturated and the signal begins to decline, the plot of which resembles a “fish-hook”, a phenomenon which is named high-dose hook effect. It was first described by Miles et al (14) with a two-site IRMA test for ferritin. It was then described in many hormones such as prolactin, growth hormone, TSH, thyroglobulin, gonadotropins (LH and FSH), testosterone, hCG, calcitonin, and aldosterone. The high-dose hook effect can be averted by a change of the sample antigen to reagent antibody ratio either by assay reformulation or dilution of the sample. To our knowledge, this is the first report of high-dose hook effect detected in 17-OHP assays.

Another interesting aspect of the present case was the development of breast tissue after the initiation of glucocorticoid therapy. It is well-known that estrogens stimulate, while the androgens inhibit breast development independently of gender. Androgen excess due to an adrenal tumor or adrenal hyperplasia suppresses normal breast development in girls, despite apparently adequate estrogen levels (15). However, in the present case, there was no breast development in addition to a very low estrogen level. It has previously been reported in rat studies that dihydrotestosterone, a 5α reduced metabolite of testosterone, has been reported to act as a competitive inhibitor of aromatase activity in the ovary (16). Therefore, in this case, a very high level of testosterone might possibly cause inhibition of aromatase activity which in turn blocks the estrogen production and subsequently breast development. After initiation of glucocorticoid therapy, breast development has begun, along with a decrease in testosterone level and an increase in estrogen level. Further studies are needed to support and verify this hypothesis.

In conclusion, to our knowledge, this is the first case report of hook effect for 17-OHP immunoassay in a patient with 21-OHD. High-dose hook effect should be suspected in patients with CAH when the test results are incompatible with one another. Additionally, this case is unique in a way that it demonstrates that a high testosterone level can block aromatase activity and therefore estrogen production and breast development.

## Figures and Tables

**Table 1:**
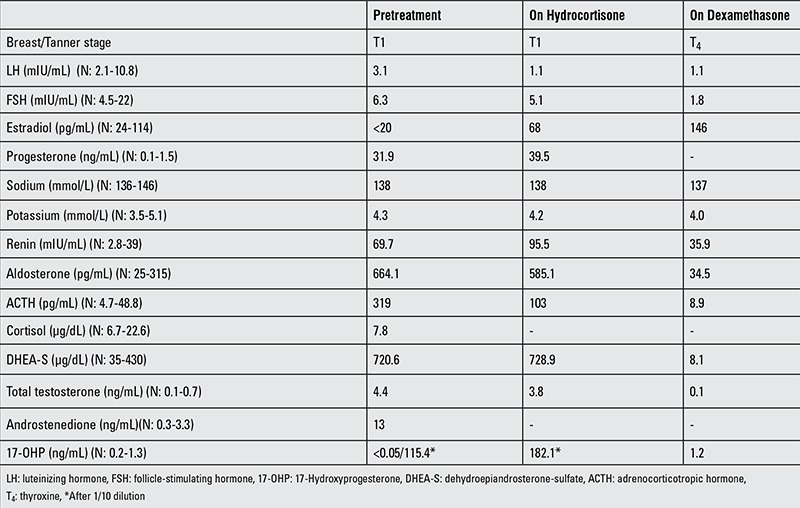
Clinical and biochemical findings of the patient
